# Mental health markers and protective factors in students with symptoms of physical pain across WEIRD and non-WEIRD samples – a network analysis

**DOI:** 10.1186/s12888-024-05767-3

**Published:** 2024-04-24

**Authors:** Tanya Tandon, Mayron Piccolo, Katharina Ledermann, Richard J. McNally, Rashmi Gupta, Naser Morina, Chantal Martin-Soelch

**Affiliations:** 1 Unit of Clinical and Health Psychology, University of Fribourg, Rue de Faucigny 2, CH-1700 Fribourg, Switzerland; 2 Department of Psychology, Harvard University, Cambridge, USA; 3 Cognitive and Behavioural Neuroscience Laboratory, Department of Humanities and Social, Sciences, Indian Institute of Technology Bombay, Mumbai, India; 4 Department of Consultation-Liaison Psychiatry and Psychosomatic Medicine, University Hospital Zurich, University of Zurich, Zurich, Switzerland

**Keywords:** Physical pain, Mental health markers, Protective factors, Network analysis, University students

## Abstract

**Background:**

Studies conducted in Western societies have identified variables associated with chronic pain, but few have done so across cultures. Our study aimed to clarify the relationship between specific mental health markers (i.e., depression, anxiety, posttraumatic stress disorder [PTSD], perceived stress) as well as specific protective factors (i.e., social support and self-efficacy) related to physical pain among university students across non-WEIRD and WEIRD samples.

**Method:**

A total of 188 university students (131 women and 57 men) were included in the study. We used network analysis to ascertain mental health markers especially central to the experience of physical pain.

**Results:**

No statistically significant difference was found between mental health markers (i.e., depression, anxiety, perceived stress, and PTSD) and protective factors (i.e., social support and self-efficacy) associated with physical pain symptoms for Swiss students versus Indian students (*M* = 0.325, *p* = .11). In addition, networks for Swiss versus Indian students did not differ in global strength (*S* = 0.29, *p* = .803). Anxiety was the most central mental health marker, and social support was the most important protective factor related to physical pain in both countries. However, for Swiss students, perceived stress, and for Indian students, PTSD symptoms were central mental health markers related to physical pain.

**Conclusion:**

Our results identify factors that may serve as important treatment targets for pain interventions among students of both countries before it becomes chronic.

## Introduction

More than 20% of youths experience a mental health disorder by the end of adolescence and 45% of the global burden of disease lies in the youth age range (18–25 years). These issues have now increased due to the stress of the COVID-19 pandemic especially among students [1]. In addition to mental health issues, physical pain has become a major health issue in this population. For instance, around 54% of university students report physical pain each year worldwide [2]; 49% in Switzerland [3], 66.9% in the United Kingdom [4], 17.5% (wrist/hand pain) to 31.4% (neck pain) in the Netherlands [5], and up to 81% in India [6]. Previous studies evidenced that physical pain leads to lower quality of life and reduced general work productivity among university students [7] and impairs their reward processes and the motivation to obtain a reward, which eventually weakens their academic performance [8]. If the reward process is disrupted in students because of pain, they might also become predisposed to a greater probability of developing a disorder, or this could serve as a mutual maintenance factor for psychopathological symptoms in the future [9]. This highlights the fact that there is an urgent need to understand factors that maintain the high frequency of physical pain symptoms in university students that could be extended to the youth population in general.

Many mental health markers and protective factors have been identified that are associated with physical pain experienced by university students. For instance, undergraduate students in Canada suffering from lower back pain (*N* = 1013) experienced high rates of depression [10]. Similarly, a study conducted on adults (*N* = 655) showed positive associations between exposure to trauma and somatic symptoms, such as pain [11]. Similarly, other studies showed a strong association between depressive and anxiety symptoms [12], as well as higher perceived stress [13] in people diagnosed with chronic pain and/or reporting higher pain severity. In summary, these studies suggest that depression, posttraumatic stress disorder (PTSD), anxiety, and perceived stress may function as risk factors for the development of pain symptoms. On the other hand, less is known about the protective factors that can help decrease the likelihood of physical pain. Protective factors are very important as the absence of risk factors does not predict a successful adaptation to the pain [14]. Therefore, focusing on protective factors might help manage physical pain and promote successful adaptation. In people with pain, higher self-efficacy and social support acted as protective factors and were related to reduced pain intensity [15, 16]. For instance, a study conducted on women (*N* = 82) living in Italy and experiencing chronic pain showed that higher self-efficacy and social support from family and friends led to decreased levels of pain symptoms [17].

Understanding risk and protective factors may help reduce pain symptoms in students around the world, however, most studies have been conducted in Western, educated, industrialized, rich, and democratic (WEIRD) societies, particularly in European countries and the United States, while only a few studies on physical pain in students were conducted in Asia, specifically Southeast Asia, despite its equally high prevalence. In that framework, a recent study conducted by our group indicated that pain impairs reward processing [3], and it can vary across cultures [18]. For example, investigating a sample of Swiss university students, we found a significant relationship between mood ratings and monetary reward in participants without subclinical pain symptoms compared to those with subclinical pain symptoms [i.e., university students with non-chronic yet clinically significant pain symptoms based on the cut-off from the pain subscale of Symptom Checklist-27-plus [3, 19]. However, when replicating the same study in a non-WEIRD sample, i.e., in India, we did not see the effect of subclinical pain on reward processes in the Indian students, although pain symptoms were reported in both samples [18]. This might be explained as the experience of pain and pain-related impairments differ across cultures, underscoring the need to expand research to non-Western cultures.

Taken together, the intercultural differences and similarities between the mental health markers and protective factors associated with physical pain among students from WEIRD and non-WEIRD samples are less known. Prior studies conducted in Western societies identified specific factors, i.e., depression, anxiety, perceived stress, PTSD, and protective factors like social support and self-efficacy associated with physical pain. However, there may be differences between countries, such as India and Switzerland. No studies have investigated mental health markers and protective factors associated with physical pain symptoms in these samples. Accordingly, we aimed to clarify the possible differences and/or similarities across non-WEIRD and WEIRD samples in the interaction between mental health markers (i.e., depression, anxiety, PTSD, perceived stress) and protective factors (i.e., social support and self-efficacy) and physical pain among university students. We used network analysis samples of Swiss and Indian university students. So far, to our knowledge, no network analysis has been conducted in this field. In addition, given the exploratory nature of the present study, no specific hypotheses were formulated.

## Method

### Participants

Participants were recruited through flyers and emails from four universities in India and three universities in Switzerland. In the flyer and emails, we asked the participants if they have a good command of English for India and French for Switzerland, if they are a university student between 18 and 25 years of age (Bachelor/Master) and if they are willing to participate in an online study investigating the relationship between psychopathology and the reward-related processes which would require them to complete online questionnaires that will take 45 minutes and perform on an online experimental task that will take 20 minutes and depending on their performance, they will have a chance to gain monetary reward (Rs 120 for Indian students and CHF 12 for Swiss students). General inclusion criteria were that students should be 18–25 years of age and have a good command of English for India and French for Switzerland. Table 1 provides the participants’ demographics. Around 200 students agreed to participate, and 188 students (131 women and 57 men) were included in this study, reflecting the participation rate of 94% of the total sample as 6% of the students started the study but did not finish it. Of those, 87 students (*M*_age_ = 21.77 years, *SD* = 2.31; 50% Females) were from India, and 101 students were from Switzerland (*M*_age_ = 21.75 years, *SD* = 3.81; 87% Females). The mean age of the total sample was 21.77 years (SD = 3.22 years). Most participants studied psychology (67%) and the other fields of studies were engineering (4.7%), natural sciences, journalism, geography, biotechnology, and political science and they were all enrolled as a student at the time of the recruitment process. There are currently no clear guidelines for sample size requirements in network analysis [20] but we based our sample size on a network analytic study of the causes of low back pain [21] that used a sample size of 123.

**Table 1:**
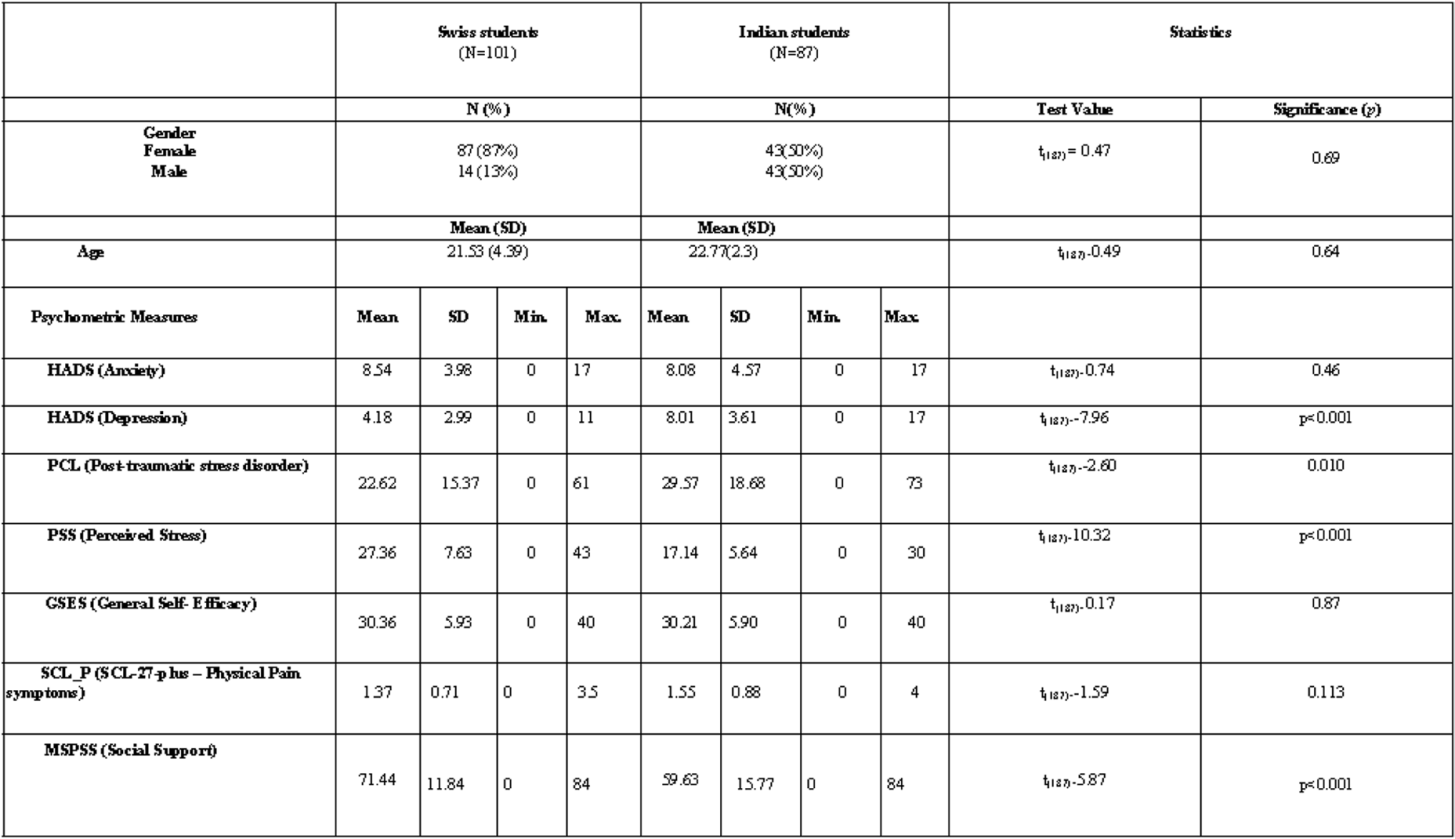
This table provides the participants’ sociodemographic characteristics and the clinical scores of students from both countries (India and Switzerland) on different psychometric measures used in the study

### Procedure

An online link to the survey was sent to students from universities in Switzerland and India. Online informed consent was obtained from all participants. Participants were allowed to terminate the survey at any time they desired. The survey was anonymous, and the confidentiality of information was maintained. Data were collected from April 2020–April 2021 via the LimeSurvey® software (LimeSurvey GmbH, Hamburg, Germany. URL http://www.limesurvey.org).

### Psychometric measures

#### Symptom checklist (SCL-27-plus)

The Symptom Checklist (SCL-27-plus [19];) is a multidimensional assessment instrument for mental health status. With 27 items rated on a 5-point Likert-type scale from 0 “never” to 4 “very often”, it consists of five dimensions: depression (5 items), vegetative symptoms (5 items), agoraphobic (4 items), and social phobia (5 items) and pain symptoms (6 items). A lifetime assessment for depressive symptoms (5 items) and screening questions for suicidality (3 items) are also included. The global score can range from 0 up to 100. This scale measures the momentary pain by using the cut-off score specified in manual [19]. This scale measures the perception of physical pain during the last 2 weeks. In the pain subscale, participants rated the following symptoms: headaches, chest pain, muscle cramps, muscle aches, arm/leg pain, and lower back pain for 0 “*never*” to 4 “*very often*” on a pain subscale depending on how often these symptoms occur in the past 2 weeks. A value of 0 stood for “never”, 1 stood for “1–2 days”, 2 for “3–7 days”, 3 for” 8–12 days”, and 4 for “13–14 days”. The overall Cronbach’s alpha coefficient for the Swiss sample in this study was 0.92 and for the Indian sample, it was 0.87.

#### Hospital anxiety and depression scale (HADS)

The Hospital Anxiety and Depression Scale (HADS [22, 23]) is a self-assessment screening scale for depression and anxiety symptoms. It consists of a 14-item scale (7 relating to anxiety symptoms and 7 to depression); each item is coded 0 to 3. The total score can range from 0 to 42. The clinical cut-off score on depression or anxiety scales is equal to or greater than 11. The overall Cronbach’s alpha coefficient for the Swiss sample in this study was 0.91 and for the Indian sample, it was 0.86.

#### Perceived stress scale (PSS)

The Perceived Stress Scale (PSS [24];) is a well-validated instrument for assessing the degree to which situations in one’s life are appraised as stressful. There are 14 items on a 5-point Likert-like scale from 0 “never” to 4 “very often”, designed to tap into how unpredictable, uncontrollable, and overloaded respondents find their lives. A total score is calculated by adding the 14 items (0 to 56), with higher scores indicating higher levels of perceived stress. A total score higher than 20 indicates high perceived stress and a total score less than 20 indicates low perceived stress on this scale. The overall Cronbach’s alpha coefficient for the Swiss sample in this study was 0.70 and for the Indian sample, it came out to be 0.72.

#### Posttraumatic stress disorder checklist (PCL-5)

The PTSD Checklist (PCL-5, [25]) is a self-report rating scale for assessing post-traumatic stress disorder (PTSD). The PCL-5 is a 20-item self-report measure designed to assess the DSM–5 symptoms of PTSD. For each symptom, respondents provide a severity rating ranging from 0 to 4 that indicates the degree of distress associated with each symptom (0 “not *at all”* to 4 “*extremely”)*. PTSD symptoms severity is measured by summing scores across the 20 items. A total score ranges from 0 to 80. The cutoff score between 31 and 33 is indicative of probable PTSD. The overall Cronbach’s alpha coefficient for the Swiss sample in this study was 0.70 and for the Indian sample, it was 0.87.

#### Multidimensional scale of perceived social support (MSPSS)

The Multidimensional Scale of Perceived Social Support (MSPSS, [26]) measures perceived social support and is composed of 12 items that cover three dimensions: Family, Friends, and Significant others. The items are rated on a 7-point-Likert scale ranging from 1 = “very strongly disagree”; 7 = “very strongly agree”. A total score is calculated by summing all items: the higher the score the higher the perceived social support. Scores for each scale can be also calculated. The overall Cronbach’s alpha coefficient for the Swiss sample in this study was 0.78 and for the India sample, it was 0.82.

### Statistical analysis

Network analysis approaches to ascertain relations between symptoms, resulting in a network of connected symptoms that activates or influences symptoms, which in turn promotes the activation of other symptoms in a cascading system leading to the onset of a mental disorder [27]. Identifying the strength of association between the specific factors and physical pain through a network approach and knowing the possible differences across two different cultures will hopefully inform culturally sensitive interventions. Given the exploratory nature of the present study, no specific hypotheses were formulated.

### Network estimation

We estimated a partial correlation network between mental health markers and protective factors associated with physical pain symptoms in university students in India and Switzerland. Data were inputted into JASP (Version 0.14.1.0), a statistical software package (https://jasp-stats.org), with analyses written in either R or C++ used in the study to conduct the network analysis [28]. Networks were created using the *qgraph* [29] R-package. With this package, a partial correlation network was created using the Extended Bayesian Information Criterion Graphical Least Absolute Shrinkage and Selector Operator (EBICglasso) method, an operation adjusted from the Least Absolute Shrinkage and Selector Operator (LASSO) regularization method [30]. Regularization retains the interitem partial correlations with the greatest magnitude while shrinking trivially small ones to zero. The hyperparameter, which determines the degree of shrinkage, was set to 0.5, the default for EBICglasso on excluding spurious edges [28]. These networks can be visualized such that nodes (mental health markers and protective factors) appear as circles connected by lines representing the edge weights (i.e., the partial correlation between individual nodes). Thicker lines represent a stronger absolute magnitude of the partial correlation, with blue lines representing positive associations and red lines representing negative associations.

### Centrality

Centrality indicates the extent to which any given node (mental health markers and protective factors) is important in the overall network. We examined *strength centrality* for each node within the Swiss and Indian networks, representing the sum of absolute edge weights connecting that node to all other nodes in the network. We used *expected influence* centrality – the sum of edges connected to a node that considers the sign of the partial correlation (i.e., either positive or negative) [31].

### Network comparison

Lastly, we compared mental health markers and protective factors with symptoms of physical pain networks between Indian students versus Swiss students by using the *NetworkComparisonTest* (NCT) package in R [32] using 1000 iterations. NCT compared networks, estimated with EBICglasso [29], on network invariance and global strength. Network invariant structure (*M*) represents the extent to which the structure of the network (i.e., distribution of edge weights) is identical across groups (i.e., Indian versus Swiss students). Global strength (*S*) represents the extent to which the overall connectivity among nodes is similar across groups, regardless of similarities or dissimilarities in network structure.

## Results

Descriptive statistics for the network variables are presented in Table 1. Indian students (*N* = 87) versus Swiss students (*N* = 101) did not differ in age (t _(187)_ = 0.49, *p* = 0.64) and gender (t _(187)_ = 0.47, *p* = 0.69). Pain scores did not differ between the groups (*p* = .11). However, a statistically significant difference was seen for depression (t _(187_) = − 7.96, *p* < .001), perceived stress (t _(187)_ = 10.32, *p* < .001), and social support (t _(187)_ *=* 5.87, *p* < .001), between the two groups. We found that Swiss students had higher stress levels than Indian students, whereas Indian students reported higher levels of depression than Swiss students. Perceived social support came out to be higher in Swiss students than in Indian students.

### Graphical LASSO and expected influence centrality

**Fig. 1:**
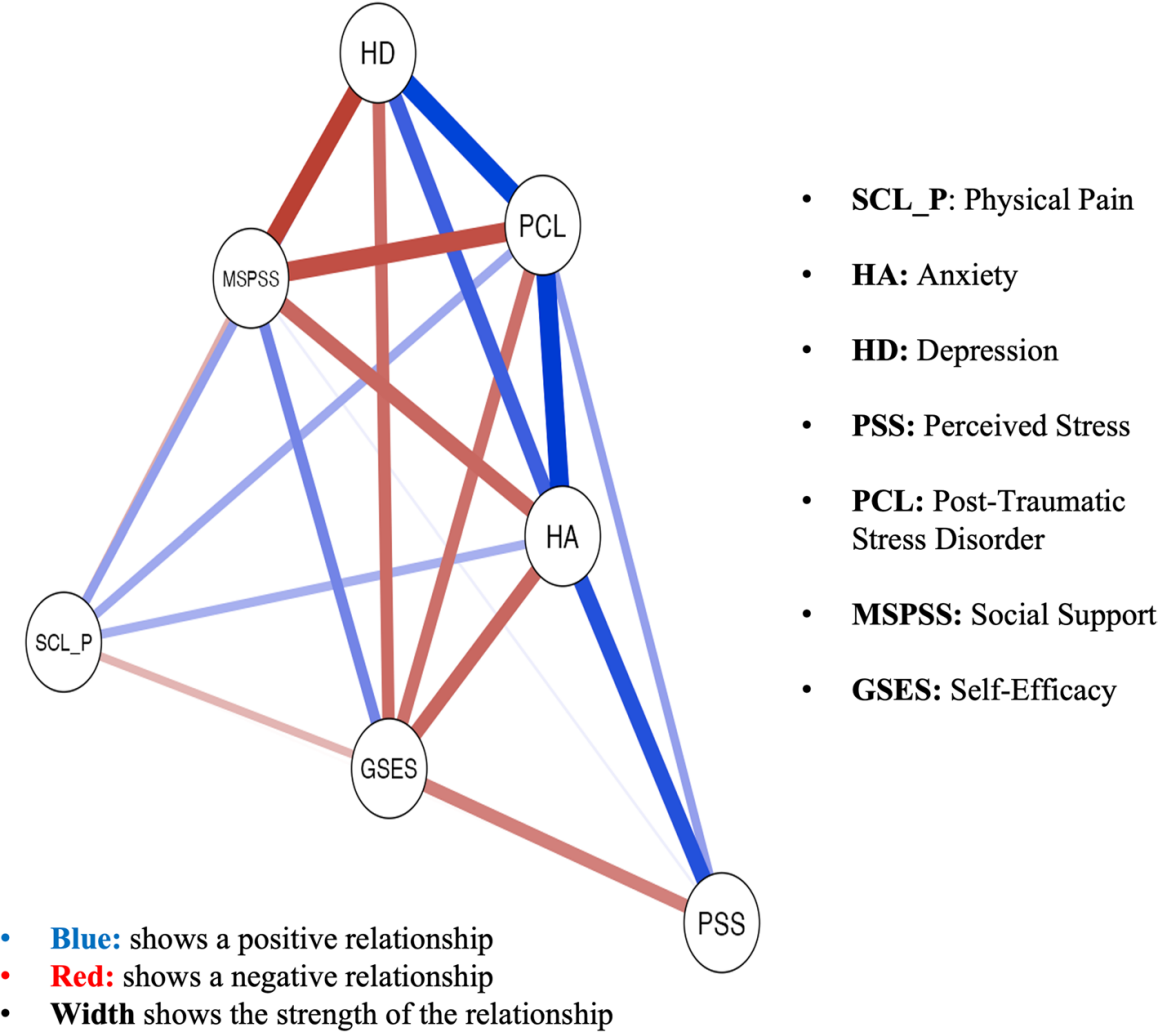
This figure shows the regularized partial correlation networks for the whole sample (India and Switzerland)

**Fig. 2:**
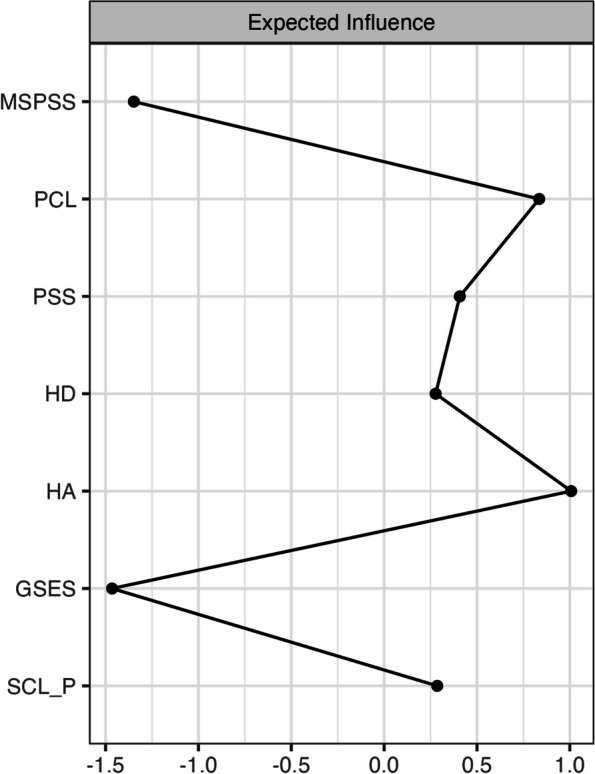
This figure shows the expected influence centrality indices which indicate the most important nodes for both samples were anxiety (HA), post-traumatic stress disorder (PCL), perceived stress (*PSS*), and depression (HD)

**Fig. 3:**
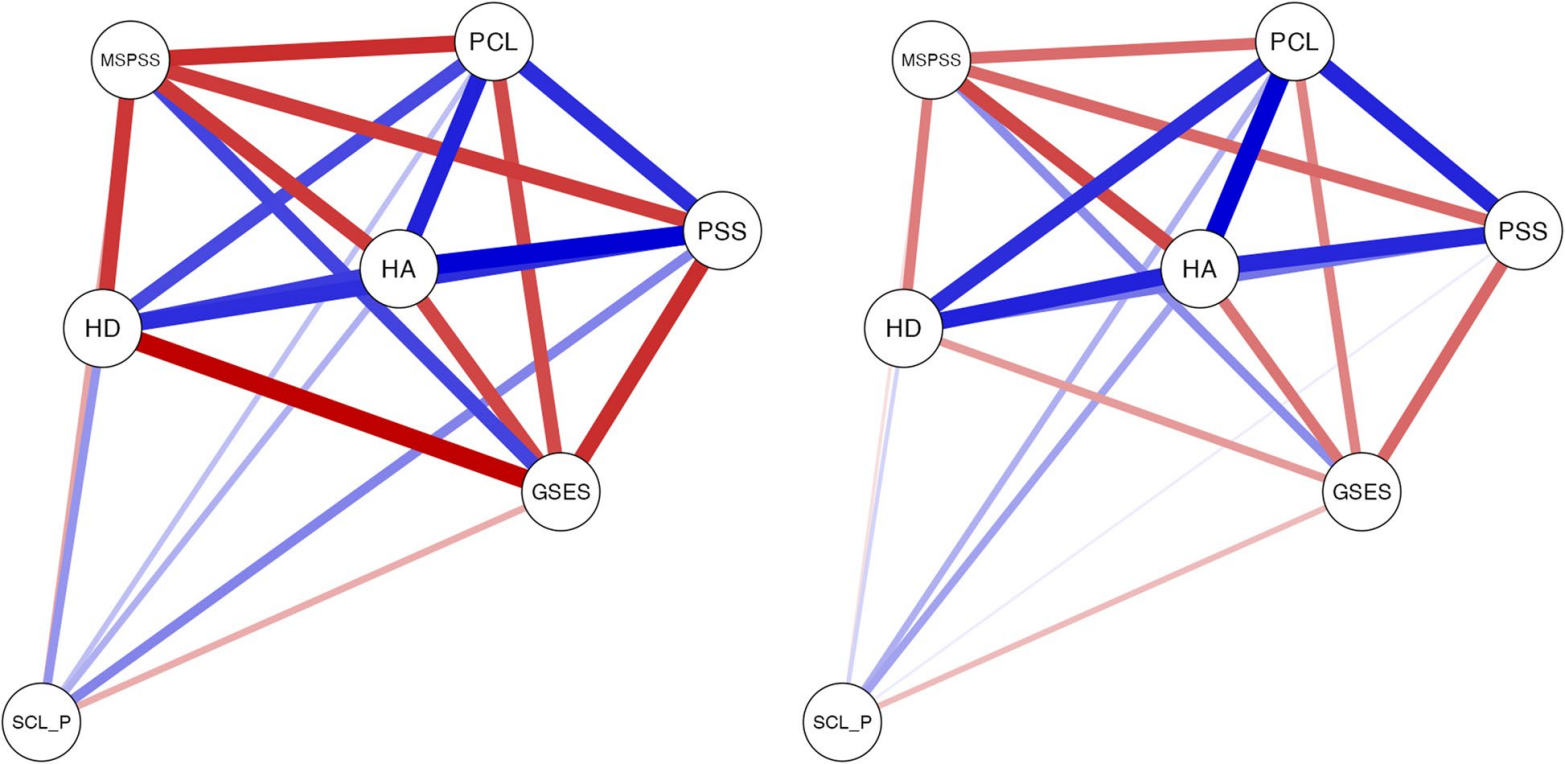
These figures show the regularized partial correlation networks for Swiss (left) and Indian students (right). *Note:* Anxiety *(HA)*, post-traumatic stress disorder *(PCL)*, perceived stress (*PSS*), depression *(HD)*, post-traumatic stress disorder *(PCL)*, and social support *(MSPSS)*, Pain *(SCL_P)*

**Fig. 4:**
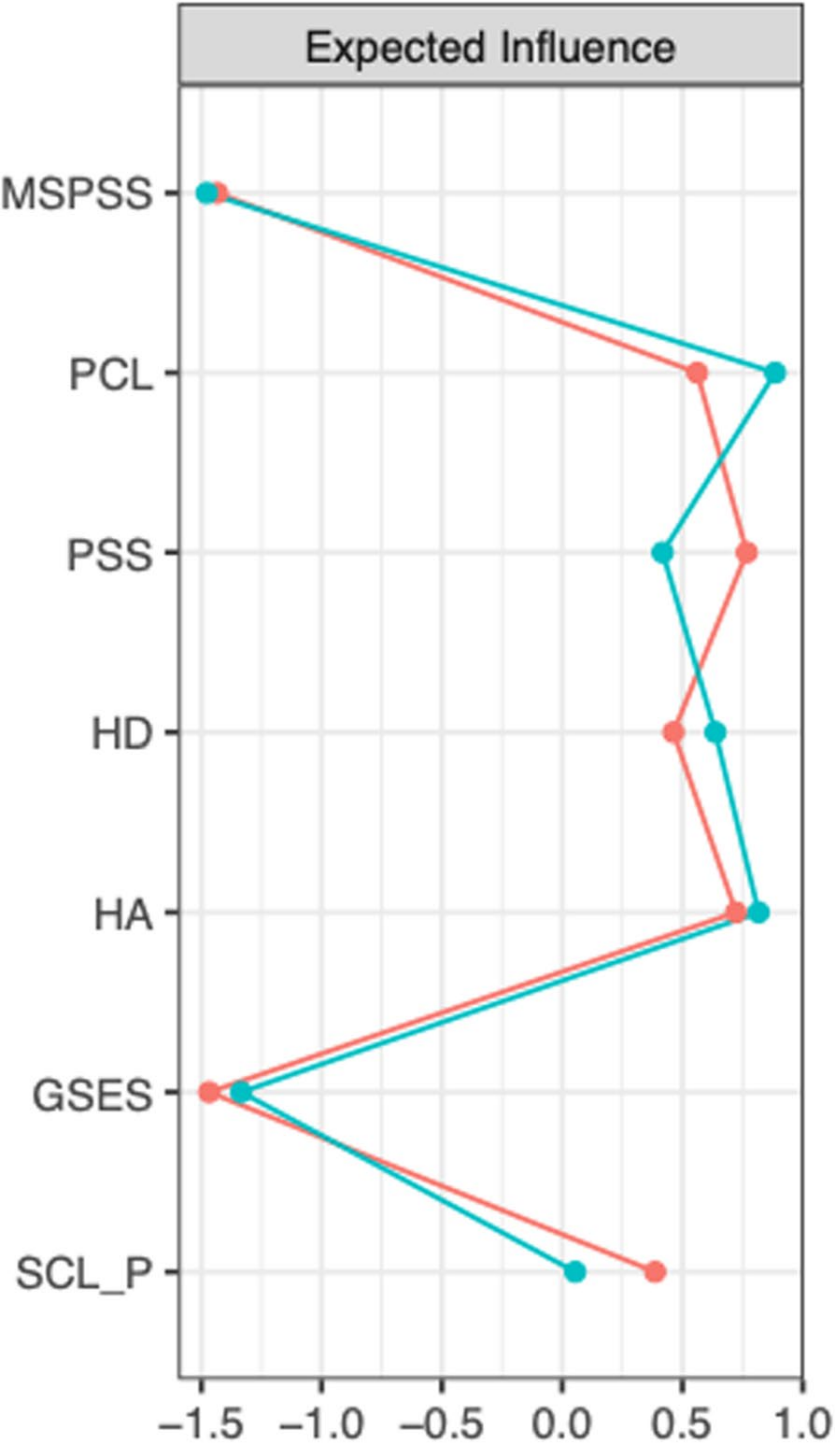
This figure shows the expected influence centrality indices which indicate the most important nodes for Swiss samples (red) were perceived stress (*PSS*), anxiety (HA), post-traumatic stress disorder (PCL), and social support (MSPSS), while for the Indian sample (blue), the most important nodes were post-traumatic stress disorder (PCL), anxiety (HA) and depression (HD) and self-efficacy (GSES)

Regularized partial correlation networks for the whole sample are presented in Fig. 1. Expected Influence centrality indices (Fig. 2) indicated the most important nodes for both samples were anxiety (HA), post-traumatic stress disorder (PCL), perceived stress (*PSS*), and depression. Figure 3 shows the regularized partial correlation networks for Swiss (left) and Indian students (right). Expected Influence centrality indices (Fig. 4) indicated the most important nodes for Swiss samples were perceived stress (*PSS*), anxiety (HA), post-traumatic stress disorder (PCL), and social support (MSPSS), while for the Indian sample, the most important nodes were post-traumatic stress disorder (PCL), anxiety (HA) and depression (HD) and self-efficacy (GSES).

### Network comparison test

NCT results indicated that no statistically significant difference was found between mental health markers (i.e., depression, anxiety, perceived stress, and PTSD) and protective factors (i.e., social support and self-efficacy) associated with physical pain symptoms for Swiss students versus Indian students (*M* = 0.325, *p* = .11). Networks for Swiss versus Indian students did not differ in global strength (*S* = 0.29, *p* = .803).

## Discussion

This study aimed to explore the interaction between specific mental health markers and specific protective factors correlated with physical pain in university students as well as to elucidate the possible similarities and differences across non-WEIRD and WEIRD samples in India and Switzerland. To our knowledge, this is the first study to test for possible similarities and differences using a network analysis approach.

Interestingly, the exploratory analysis revealed that the two countries showed significant statistical group differences regarding perceived stress, depression, and social support. Surprisingly, our study found that perceived social support (i.e., the resources perceived as being available from others like family and friends in our social networks) seemed higher in Swiss students than in Indian students. This contradicts the intuitive prediction based on the relationships between WEIRD and non-WEIRD countries. This counterintuitive cultural pattern may be explained as, in Western countries, relationships are seen as promoting individual goals. For instance, one may seek help from their immediate environment to achieve personal goals [33, 34], whereas in collectivistic cultures, a person is fundamentally connected to others, and the emphasis is placed on group harmony, and any efforts made to bring personal problems to the attention of the others may harm the group harmony [35]. This might lead people from non-WEIRD societies not to seek help from their immediate environment. One of the studies conducted on Korean students (*N* = 56) and American students (N = 56) showed similar results. American students were more likely to mention using social support than Koreans [36]. However, in the current study, we reported the differences in the total MSPSS score [26]. However, additional analyses, not reported in this paper’s objective, showed that when the dimensions of the MSPSS were used, the difference between Switzerland and India in family support was nonsignificant, whereas the differences in friend and significant others support were significant. Thus, it may be possible that the groups in which the support is perceived make a difference, and it may be important to consider them, especially in cross-cultural studies. Also, our study found that Swiss students had higher stress levels than Indian students, whereas Indian students reported higher levels of depression than Swiss students. This highlights that mental health is a major health concern in developing and developed nations, although mental health markers might differ across countries. Also in our study, we found a strong positive relationship between mental health markers (i.e., depression, anxiety, perceived stress, and PTSD) with physical pain. This shows that mental health markers might make a person vulnerable to developing chronic pain in the future, which aligns with many previous studies [37–39]. In addition to this, we also found protective factors (i.e., self-efficacy and social support) were negatively related to physical symptoms of pain, which shows that higher levels of protective factors might be associated with a reduced likelihood of experiencing physical symptoms of pain and might lead to better daily physical function and a better quality of life in the lives of the student [40].

The results of our network analyses reveal the association between stress symptoms, PTSD symptoms, anxiety, and depression appeared to be particularly important for physical pain in both countries in students. The edges connecting these three mental health markers (i.e., stress, PTSD, anxiety, and depression) were among the strongest edges of the network, indicating that the association between these symptoms is a core feature of physical pain across two countries. Notably, interventions designed to weaken the association between these three mental health markers in students might decrease physical pain symptoms. However, there were no significant differences between the two countries’ network structure and global strength. Anxiety was the most central symptom for both countries as indexed by the magnitude of association with physical pain, consistent with other research on young adults (18–25 years old) [41–44] and anxiety might be an important factor in predicting youth who are at greatest risk for increased impairment because of pain symptoms [45]. Indeed, anxiety is emerging as a potent risk factor for physical pain risk in young adults [46]. Several human brain imaging studies have provided better support for clinical observations of the interaction between pain and anxiety and showed that anxiety enhances the experience of pain [44, 47, 48]. This suggests potential targets for treating anxiety. In addition, social support was the important protective factor negatively related to physical pain in our network for both countries. Many studies have shown that people with chronic pain who report high levels of social support experience less distress and less severe pain, with higher levels of support associated with better adjustment in daily life despite the pain-related challenges [49, 50].

Interestingly, PTSD seemed to be the most central mental health marker related to physical pain symptoms in the network for Indian students. Previous studies have revealed that exposure to traumatic events is quite prevalent in India [51, 52], with findings from the largest-ever representative survey [53] of the prevalence of child abuse and neglect in India showing how 2 out of every 3 youth have experienced physical abuse, sexual or emotional abuse once in their lives. One study conducted by Bhat and Rangaiah [54], showed that 49.81% of young adults (19–24 years) (*n* = 797) encountered at least one traumatic event, with the most common event being the death of a close one, serious illness, witnessing the injury or killing of others, and coming close to being injured or killed. Substantial literature has revealed that traumatic events are one of the risk factors leading to chronic pain [55–57]. In one of the studies conducted in the Norway reported that the exposure to traumatic events and PTSD were significantly associated with more severe physical pain, and PTSD significantly moderated the relationship between trauma exposure and pain [58] and in one of the cohort studies that included 2021 participants from the USA observed over time that after traumatic stress exposure, identified individuals with greater pain severity [59]. In our study, this was shown in the network of Indian students suggesting a potential target for an intervention designed for young people in India for the prevention of issues related to physical pain that might become chronic in the future. Perceived stress is quite common among the university students, in Malaysia 38% of the students [60], in Saudi Arabia 52% of the students [61] and among Italian students, 8 to 31.4% [62] suffers from it. In Swiss students, stress was the most important mental health marker. Stress is quite commonly seen in university students as they are in a major transitional phase of moving out, managing their household, financing, and becoming independent from their parents [63]. This puts a lot of pressure on them leading to social, emotional, and academic challenges. Roughly half of Swiss students, 46% of them suffer from perceived stress in which personal resources are no longer able to address daily challenges [64]. One of the systematic reviews found that stress plays an important role in the development of pain-related issues in young adults [65], consistent with our findings. Our study suggests that stress would be a potential target for an intervention designed for young people in Switzerland for the prevention of issues related to physical pain that might become chronic in the future.

Our study highlights the central symptoms and associations related to physical pain and provides us with an important direction to examine the dynamics of activating and deactivating those central mental health markers and protective factors and associations [66]. For example, potential treatments that deactivate a central symptom or association could lead to the spreading deactivation of other less central elements and be more effective in treating physical pain-related issues among students before they become chronic [67]. So, interventions that specifically target stress, PTSD, anxiety, and depression might efficiently and effectively reduce physical symptoms, increase the likelihood of social support, and possibly reduce the risk of relapse. However, these possibilities remain open to empirical questions. Also, these symptoms and associations between symptoms can be prioritized in theoretical models of physical pain and could also serve as important treatment targets for pain interventions among students before it becomes chronic in the future for both countries. Katz and Selzer [68] mentioned that the transition from acute to chronic pain may reveal important cues that predict whose acute pain will become chronic. However, Lee et al., [69] showed that factors like social support that promote pain adaptation can protect the individual from transitioning to chronic pain from acute pain. Based on our study, we can identify those specific risk factors and protective factors for Indian students and Swiss students and target those factors in the future studies.

The above results also make us question the fact that what could be the reason behind the similarities of the networks in the two countries though keeping in mind that our sample represents the part of the country. This might be explained as many studies have focused primarily on East–West differences in individualism–collectivism or independent–interdependent constructs though these are important [70]. At the same time, we forget that we live in a globalized world. According to Ralston [71], there is a “cross-vergence” of cultures which means there is a shortening of cultural distances between countries. Also, our sample comprised a young population (18–25 years of age) who uses social media the most, and due to the convergence of social media and globalization, the world has shrunk into a much smaller interactive field [72]. Social media has brought people from different cultures together in the “global village” [73]. Young people use social media to learn about other countries, establish and maintain relationships, and stay informed about events happening in different countries [74]. Therefore, it leads to broad similarities in underpinning risk and protective mechanisms related to mental health, although these are often influenced by cultural and other contextual factors that might differ across or within countries [75].

Several limitations deserve mention. First, self-report instruments may underestimate symptom severity in cultures that stigmatize psychopathology. Second, the cut-off used in the study was for a Western population, which might not be representative of Indian reality and a lack of validation of instruments in the Indian population. Future studies should seek to validate these instruments in Indian and non-WEIRD samples and determine specific cut-offs for these populations. Third, our study only represents a part of the country, and it might be helpful to include samples from different regions within the same country to first understand the within-country differences and similarities and then expand it further across different countries.

In conclusion, our findings provide promising evidence of mental health markers and protective factors related to physical pain in the two countries and some cultural variations between them. Our study is the first to explore this relationship using the network analysis approach. This relationship with the university students provides the first insight into the development of culturally specific preventive interventions in students.

## Data Availability

The datasets used and/or analysed during the current study available from the corresponding author on reasonable request.
